# Optical Fiber‐Assisted Printing: A Platform Technology for Straightforward Photopolymer Resins Patterning and Freeform 3D Printing

**DOI:** 10.1002/advs.202403049

**Published:** 2024-06-21

**Authors:** Alessandro Cianciosi, Maximilian Pfeiffle, Philipp Wohlfahrt, Severin Nürnberger, Tomasz Jungst

**Affiliations:** ^1^ Department for Functional Materials in Medicine and Dentistry Institute of Functional Materials and Biofabrication University of Würzburg and KeyLab Polymers for Medicine of the Bavarian Polymer Institute (BPI) Pleicherwall 2 97070 Würzburg Germany

**Keywords:** alkyne‐modified gelatin, allyl‐modified gelatin, optical fiber‐assisted printing, photoclick resins

## Abstract

Light‐based 3D printing techniques represent powerful tools, enabling the precise fabrication of intricate objects with high resolution and control. An innovative addition to this set of printing techniques is Optical Fiber‐Assisted Printing (OFAP) introduced in this article. OFAP is a platform utilizing an LED‐coupled optical fiber (LOF) that selectively crosslinks photopolymer resins. It allows change of parameters like light intensity and LOF velocity during fabrication, facilitating the creation of structures with progressive features and multi‐material constructs layer‐by‐layer. An optimized formulation based on allyl‐modified gelatin (gelAGE) with food dyes as photoabsorbers is introduced. Additionally, a novel gelatin‐based biomaterial, alkyne‐modified gelatin (gelGPE), featuring alkyne moieties, demonstrates near‐visible light absorption thus fitting OFAP needs, paving the way for multifunctional hydrogels through thiol‐yne click chemistry. Besides 2D patterning, OFAP is transferred to embedded 3D printing within a resin bath demonstrating the proof‐of‐concept as a novel printing technology with potential applications in tissue engineering and biomimetic scaffold fabrication, offering rapid and precise freeform printing capabilities.

## Graphical abstract

Optical Fiber‐Assisted Printing (OFAP) is a novel, flexible, and straightforward light‐based fabrication platform that consists of a cost‐effective LED‐coupled optical fiber (LOF). This technology allows automated LOF spatiotemporal positioning in the 3D space, thus enabling the precise 2D photopatterning and the embedded 3D printing of hydrogel‐based resins. The novel compositions of the gelatin‐based resins, tailored for OFAP, feature food dyes and alkyne moieties.

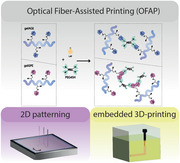

## 1 Introduction

Rapid prototyping stands as the most common strategy for the fabrication of complex 3D structures. The working principle of this fabrication process relies on the selective deposition of materials, layer‐by‐layer, which holds promise for creating 3D functional tissues with intricate and hierarchical features.^[^ ^1^, ^2^, ^3^ ^]^ There are a variety of 3D printing technologies, including extrusion‐based fused deposition modeling and inkjet‐based printing.^[^ ^4^, ^5^, ^6^, ^7^ ^]^ Despite their capability for controlled material deposition, these technologies are limited in the achievement of high resolution 3D architectures. To address these limitations, a relatively new research avenue in biofabrication is vat polymerization (VPP),^[^ ^8^, ^9^ ^]^ a form of light‐assisted printing.^[^ ^10^ ^]^ Compared to other 3D printing techniques like extrusion‐based bioprinting, VPP technologies offer notable advantages in resolution (down to the µm scale), complexity, and efficiency.^[^ ^11^ ^]^ A well‐known example of these technologies is digital light processing (DLP). DLP printers consist of a UV light source and a digital micromirror device that selectively cures, a photo‐sensitive resin with micron‐level resolution and high structural complexity and generates constructs layer‐by‐layer.^[^ ^12^ ^]^ However, to achieve high precision and highly resolved features, the size of the projection is restricted, and long printing times are required for the fabrication of large structures.^[^ ^13^ ^]^ A reduction of the printing time (<10 min for 5 mm thick constructs) has been introduced with continuous liquid interface production.^[^ ^14^, ^15^ ^]^ Nevertheless, this VPP fabrication technology is characterized by a costly and complex setup, and its efficiency is limited to the processing of low‐viscosity resins.^[^ ^13^ ^]^ The introduction of the computed axial lithography (CAL) method, inspired by computer tomography, marks additional significant progress for VPP in terms of printing times and variability of resin. CAL enables the fabrication of 3D objects with 0.30 mm features in a matter of seconds. Objects are achieved by rotating a synthetic photopolymer‐laden reservoir within a dynamic light field. Back projections of a computer‐assisted digital model accumulate a light dosage, overcoming the gelation threshold and inducing material solidification exclusively in voxels corresponding to the designed 3D construct.^[^ ^16^ ^]^ The notable potential of CAL has been demonstrated in biofabrication by volumetric bioprinting of centimeter‐scale living tissue constructs in seconds using a gelatin methacryloyl (gelMA) photoresin.^[^ ^17^ ^]^ GelMA‐based hydrogels are widely used due to their compatibility with photocrosslinking strategies that preserve their shape in physiological conditions.^[^ ^18^, ^19^ ^]^ Nevertheless, the methacryloyl moieties of gelMA undergo chain‐growth free‐radical polymerization which yields inhomogeneous polymer networks.^[^ ^20^ ^]^ Suitable alternative resins for VPP platforms using thiol‐ene click chemistry,^[^ ^21^ ^]^ which is a crosslinking approach that yields highly organized hydrogel networks.^[^ ^22^ ^]^ This polymerization strategy involves an orthogonal reaction between a carbon‐carbon double bond, which is often grafted on the gelatin backbone,^[^ ^23^ ^]^ and a thiol group of a crosslinker molecule. Furthermore, the thiol‐ene reaction features a high reaction kinetic, oxygen insensitivity, compatibility with low‐concentration monomers, and limited hydrogel shrinking and polymerization stress.^[^ ^24^, ^25^, ^26^ ^]^ Allyl‐modified gelatin (gelAGE) is an established biomaterial platform that undergoes a thiol‐ene click reaction in the presence of a thiolated crosslinker and a photoinitiator (PI).^[^ ^27^, ^28^ ^]^ VPP technologies often exploit resin formulations with photoabsorbers (PA) to attenuate the light penetration depth and to withdraw photons from the PI, thus delaying the radical formation and yielding more resolved features.^[^ ^11^, ^29^ ^]^ A common strategy for light‐based 3D printing involves incorporating PA such as non‐cytotoxic food dyes like tartrazine^[^ ^30^ ^]^ and Ponceau 4R,^[^ ^31^ ^]^ anionic azo dye,^[^ ^32^ ^]^ and 2‐hydroxy‐4‐methoxy benzophenone‐5‐sulfonic acid,^[^ ^33^ ^]^ into the resin. Similar to the thiol‐ene click reaction of gelAGE, alkynes moieties can undergo a thiol‐yne click reaction in the presence of a thiolated crosslinker. Although it was used in polymer chemistry, this crosslinking strategy has been scarcely applied in the field of biofabrication with only a few examples.^[^ ^34^ ^]^

In this study, a novel fabrication strategy named Optical Fiber‐Assisted Printing (OFAP) is introduced using a benchtop printer and a cost‐effective LED‐coupled optical fiber (LOF) to selectively crosslink photosensitive resins in 2D and 3D. The seamless integration of the LOF with an automated platform allows it to precisely control its spatiotemporal position, irradiance, and exposure time. This enables the fabrication of photopatterned structures characterized by progressive elements (e.g., star‐shaped constructs) through on‐the‐fly adjustment of their print line diameter. The developed setup is straightforward and easy to reproduce since it can be readily integrated with other automated processing technologies (e.g., robotic arms, fused filament fabrication (FFF) 3D printers), and yields micrometers‐scale resolution. The OFAP setup represents a promising alternative to the state‐of‐the‐art VPP techniques. In comparison to a standard DLP printer (Original Prusa SL1S speed), the OFAP‐based photopatterning approach showed a total fabrication rate of roughly two times faster. Furthermore, gelAGE‐based resins are developed to meet the needs of OFAP. Tartrazine (yellow food coloring FD&C Yellow 5, E102) and Allura red AC (red food coloring FD&C Red 40, E129), whose absorbance spectra cover the UV–vis light spectrum, are integrated with the resin precursor for the photopolymerization of structures with enhanced print line resolution. Moreover, this work highlights the introduction of a novel gelatin‐based resin functionalized with glycidyl propargyl ether (GPE) and featuring alkynes moieties. Additionally, the alkyne moieties show absorbance properties in the near‐visible UV range (405 nm) that enhanced the resolution of the print lines without the use of PA. It is demonstrated that the great versatility of the OFAP fabrication platform permits drawing with the LOF directly within the resin bath. As a proof‐of‐concept, the optical fiber is immersed into a gelAGE‐based resin‐laden vat and used for successful freeform 3D printing of centimeter‐scale self‐standing structures within a few minutes (3–5 min for 8 mm thick construct). The upscaling of OFAP from photopatterning to 3D printing introduces a novel printing technology for VPP. To the best of our knowledge, this work represents the first example of freeform 3D printing within a photosensitive, hydrogel‐based resin precursor. The controlled movement of the LOF within the precursor enables the fabrication of 3D self‐standing structures by exploiting the photocrosslinking reaction directly at the interface between the LOF tip and the precursor solution.

## 2 Results and Discussion

### 2.1 OFAP Photopatterning of gelAGE‐Based Resin with Real‐Time Adjustment of Fiber Resolution and Multilayer Fabrication

**Figure 1:**
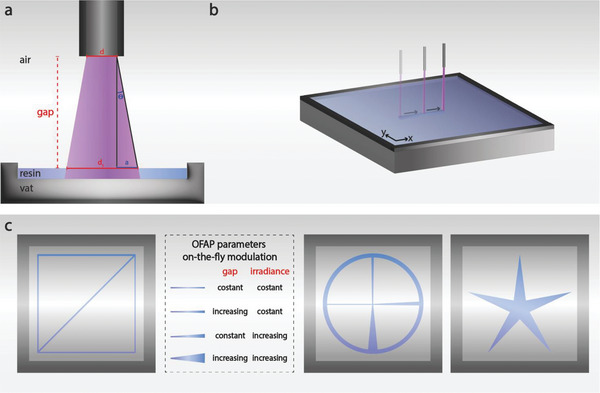
Schematic of the LOF and the OFAP photopatterning process for standard constructs and progressive elements fabrication. a) Representation of the physical parameters of the LOF and the OFAP photopatterning process. Gap: optical fiber to vat distance; d: optical fiber inner diameter; d_1_: light cone diameter at the resin‐air interface; Θ: sinus of the half‐angle of the light cone; a: widening of the light spot, at the resin surface. b) Illustration of the automated OFAP photopatterning approach. c) Schematic of the photopatterned structures and table describing the on‐the‐fly adjustments of the gap and irradiance and their influence on the print line resolution. A 10 mm‐side square design was used for the print line resolution assessment (left), a structure featuring different progressive elements, and a start‐shaped construct (right).

The OFAP photopatterning setup involves an x‐y‐z stage from a commercially available bioprinter integrated with the LOF, which perpendicularly irradiates a resin‐laden vat at a specific optical fiber‐to‐vat distance (gap) (**Figure** **1**a). The LOF consists of a quartz optical fiber featuring a core diameter (d) of 0.25 mm and a numerical aperture (NA) of 0.66. Trigonometry was used to determine the theoretical diameter of the light spot on the gelAGE‐based photoresin surface (d_1_). The electromagnetic radiation traveling through the air formed a cone with dimensions directly correlated to the NA of the optical fiber. The NA is defined as the product of the medium refractive index (air) and the sinus of the half angle of the light cone (Θ) (Equation 1).^[^ ^35^ ^]^

(1) $$\mathrm{NA}=n_{air}\times \sin\left(\theta \right)$$

The widening of the light spot in correspondence with the resin interface (a) is defined as the product of the gap and the tangent of Θ (Equation 2).

(2) $$a=\mathrm{gap}\times \tan\left(\theta \right)$$

Eventually, *d*_1_ was calculated by using (Equation 3) after introducing in Equations (1 and 2) the features of the optical fiber (*NA* = 0.66 and *d* = 0.25 mm).

(3) $$\begin{array}{ccc} d_{1} & = & d+2a=d+2\times \mathrm{gap}\times \tan\left(\arcsin\left(\frac{NA}{n_{\mathrm{air}}}\right)\right)\\ & \approx & 0.25\;\mathrm{mm}+1.76\;\mathrm{gap} \end{array}$$

Equation (3) enabled the calculation of the theoretical diameter of the light spot emitted by the OFAP platform as a function of different gap distances. Particularly, gaps of 0.05, 0.15, and 0.25 mm correspond to theoretical print line diameters of 0.34, 0.51, and 0.69 mm, respectively.

The integration of a LOF with an automated platform enabled precise spatiotemporal control for photopatterning gelatin‐based photosensitive resins (Figure 1b; Movie S1, Supporting Information). A 10 mm‐side square structure, with one diagonal, was photopatterned and used throughout the study as a standard construct to assess the OFAP printing resolution of different resin formulations (Figure 1c, left). By using a feed rate of 1 mm s^−1^ for the XY movement of the LOF, the standard 10‐mm sided square construct featuring a 0.20 mm thickness was fabricated in ≈54 s compared to the 107 s that would take using a commercially available DLP printer (Original Prusa SL1S speed). The OFAP setup allowed real‐time adjustment of variables such as irradiance and gap, thus enabling the fabrication of constructs with progressive features (Figure 1c, right).

**Figure 2:**
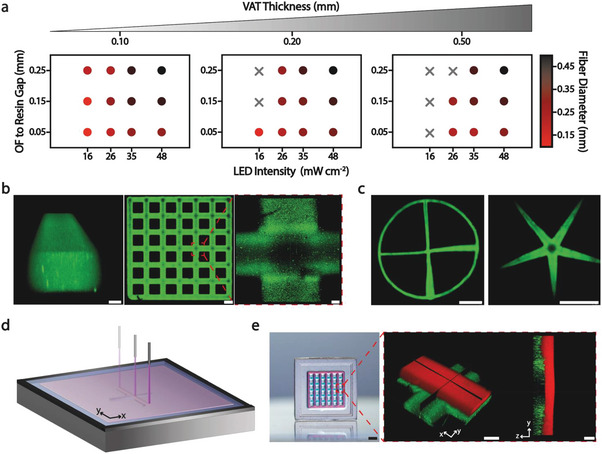
OFAP photopatterning of gelAGE‐based resins. Fabrication of lattice, and multilayer structures and constructs with incremental features. a) Interdependency of the OFAP printing process parameters (e.g., vat thickness, irradiance, gap) and their influence on the print line resolution using a gelAGE‐based hydrogel precursor (5% gelAGE, 10% PEG4SH, 0.1% LAP). One sample was analyzed at each point. Data are reported as mean values, *n* = 5 repeated measurements. b) Photopatterning of a lattice structure with a focus on the single print line (left) and their intersection (right), FITC‐dextran was used as a fluorescent dye. Scale bars (from left to right): 0.05, 0.50, and 0.05 mm. c) Photopatterned structure featuring incremental details (left) and a star‐shaped geometry (right). Scale bars: 3 mm. d) Schematic of the OFAP multilayer fabrication. e) Multilayer gelAGE‐based lattice structure fabricated with two adjacent layers stained with fluorescent dyes (green: FITC‐dextran, red: Texas red‐dextran) characterized by distinct excitation spectrum. Scale bars (from left to right): 3, 0.10, and 0.20 mm.

OFAP photopatterning was focused on spatially controlled irradiation. The first step in this study was to investigate the effects of the physical parameters of the OFAP setup on the construct resolution. A holistic approach was used to understand the interplay of the OFAP photopatterning features as gap, vat thickness, and irradiance and their effects on the print line resolution (**Figure** **2**a). The initial hypothesis based on the theoretical light spot diameter values indicated a linear correlation between the increasing gap distances and the construct resolution as a consequence of the widening of the irradiated photoresin area. Indeed, depending on the irradiance of the LED which can be adjusted between 16 and 48 mW cm^−2^ through a built‐in potentiometer, the print line diameter of the photopatterned constructs spanned from roughly 0.15 to more than 0.45 mm, while increasing the gap from 0.05 to 0.25 mm (Figure 2a). The measured print line diameters were slightly smaller compared to the theoretical ones, and this phenomenon can be attributed to the light intensity distribution emitted from the optical fiber which did not exceed the crosslinking threshold around the edges of the print lines.

Besides the gap, which influenced the diameter of the light cone as demonstrated in Equation (3), another important physical parameter that affected the photopatterning resolution of OFAP was the irradiance. A low LED intensity resulted in a reduced energy transmitted to the gelAGE‐based photoresin thus generating fewer radical species per unit of time and eventually yielding to a smaller print line diameter.^[^ ^36^ ^]^ The experimental data reported in Figure 2a confirmed the widening of the print line diameter with the increase of the irradiance. Taken together, the close intercorrelation between gap and irradiance was demonstrated to have a direct effect on the OFAP resolution while the vat thickness was also evaluated and showed a more relevant impact on the photopatterned construct stability than its resolution. The light emitted by the optical fiber traveled through the air and was refracted by the gelAGE‐based photoresin precursor. The hydrogel precursor formulation also contained a 4‐arm thiolated polyethylene glycol (PEG4SH) and lithium phenyl‐2,4,6‐trimethylbenzoylphosphinate (LAP) which is a well‐established water‐soluble PI. Nevertheless, LAP is characterized by a relatively low molar absorptivity at 405 nm (ε = 30 M^−1^ cm^−1^; 0.05 cm^−1^ absorptivity with 0.05 wt.%).^[^ ^37^ ^]^ The data reported in Figure 2a showed an increased instability of the photopatterned structure while increasing the inner thickness of the vat from 0.10 to 0.20 mm with a more evident effect when the thickness reached 0.50 mm. This phenomenon could be associated with an insufficient curing depth for low irradiance values. A similar research study conducted on gelAGE using LAP confirmed a decreased curing depth using near‐visible UV light while increasing the thickness of the photo crosslinked construct.^[^ ^38^ ^]^

A confocal image of a single gelAGE‐based print line featuring a thickness of ≈0.20 mm and a diameter of ≈0.30 mm was depicted in Figure 2b, left. Furthermore, a single‐layered lattice structure fabricated with high control, particularly on the crossover points where the irradiance overlapped, was reported (Figure 2b, right).

The initial comprehensive analysis conducted to understand the processability range of OFAP‐based photopatterning and its limits was propaedeutically used to focus on understanding the interplay between the gap and LED intensity. These OFAP physical parameters were varied during the manufacturing process to fabricate structures characterized by gradual features with high precision. Indeed, the fully automated OFAP set‐up enabled to change of the irradiance together with the optical fiber z position almost instantly, while patterning. The z resolution of the patterned structure is correlated to the curing depth which describes the thickness of the cured construct as a function of the irradiation dosage.^[^ ^39^ ^]^ The green strength of the photopatterned constructs determines their capability to withstand the curing process and remain stable. This feature is mostly correlated with the cure depth (0.05 to 0.25 mm for the OFAP photopatterning setup) and light penetration depth.^[^ ^40^ ^]^ GelAGE‐based precursor formulations using LAP as PI were already demonstrated to enable large penetration depth without hampering the photocrosslinking efficacy and stability of the cured object.^[^ ^38^ ^]^

To show the application of OFAP photopatterning for easily recreating structures with progressive elements, a design featuring four print lines perpendicularly oriented to each other and connected to the center point of the framing circle was shown (Figure 2c, left). Particularly, the print line on the left was fabricated by simply maintaining both the gap and the irradiance (average print line diameter ≈0.48 mm) constant. The print line on the top was patterned from the center point of the circle by gradually increasing the gap while maintaining the irradiance constant. In this case, only a small incremental detail (from ≈0.50 to 0.70 mm) could be noticed compared to the initial standard print line. Nevertheless, on the print line on the right, a larger increment was observed (from ≈0.60 to 1.10 mm) when the irradiance was increased while keeping the gap constant. Eventually, the combined gradual increase of the irradiance and the gap resulted in an even more noticeable progressive print line (from ≈0.60 to 1.30 mm). The stability of each gradual print line was assured as the on‐the‐fly adjustments of irradiance and gap were performed within the values reported during the initial systematic screening, using a VAT with a thickness of 0.20 mm (Figure S1, Supporting Information). Additionally, a star‐shaped photopatterned structure featuring large incremental details (from ≈1.40 to 0.10 mm) was reported (Figure 2c, right).

The OFAP set‐up was also demonstrated to fabricate multilayer structures (Figure 2d) by exploiting the thermoreversible properties of the gelAGE. In this OFAP‐based strategy, the focus was shifted from the deep understanding of the interplay between fabrication parameters, on single‐layer structures, toward exploiting only the physicochemical features of the gelAGE to obtain multilayer constructs without real‐time adjustment of fabrication parameters. Particularly, in the multilayer fabrication approach the first gelAGE‐based resin layer fluorescently stained with a fluorescein isothiocyanate (FITC)‐labeled dextran was photopatterned and the remaining precursor solution was physically cured by temperature decrease (around 4 °C). The second layer of gelAGE‐based resin containing fluorescent red‐dextran was poured on top of the first layer and photopatterned. Eventually, the non‐crosslinked precursor of both layers was rinsed to yield the final construct (Figure 2e, left). Confocal microscopy was used to highlight the lattice structure and the clear separation with the absence of mixing across the two layers (Figure 2e, right).

### 2.2 Photoabsorbers and Triple Bond Gelatin‐Grafting to Enhance the OFAP Photopatterning Resolution

**Figure 3:**
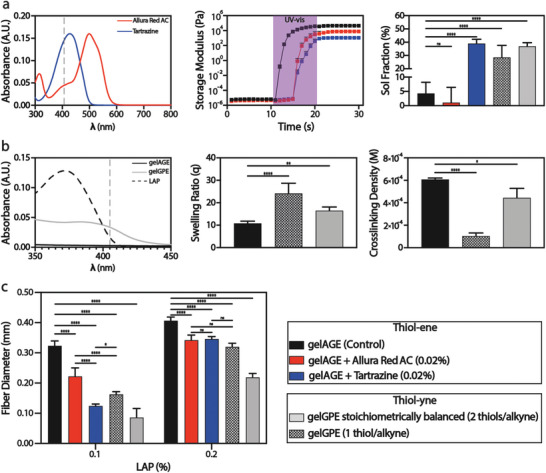
Quantitative evaluation of the effect of photoabsorbers and alkyne moieties on the print line resolution. a) Introducing PA, Allura red AC, and tartrazine, into the gelAGE‐based precursor formulation. UV–vis spectrum of both PA (0.002%, PBS as solvent, left). Photo‐rheological oscillatory analysis: Time sweeps trends of the gelAGE‐based precursor without (control) and with Allura red AC and tartrazine (center). Data are reported as mean values ± SDs, *n* = 3 independent experiments. Crosslinking efficacy expressed as sol fraction (mass loss after 24 h) of the gelAGE‐based hydrogel (control) compared to the gelAGE‐based precursors containing Allura red AC and tartrazine, respectively, and the gelGPE‐based hydrogels both stoichiometrically balanced and in thiol defect (right). Data are reported as mean ± SDs, *n* = 6 independent experiments. Statistical significances are determined based on a one‐way ANOVA. b) Absorbance properties of the gelatin featuring alkyne moieties and thiol‐yne click chemistry to tailor the hydrogel crosslinking density. UV–vis spectrum of gelGPE in comparison to gelAGE and LAP (5% of gelAGE and gelGPE, 0.1% of LAP, PBS as solvent, left). Swelling trends (after 24 h) of the gelAGE‐based hydrogels in comparison to the gelGPE‐based hydrogels both in stochiometric balance and in thiol deficit (center). Data are reported as mean ± SDs, *n* = 6 independent experiments. Statistical significances are determined based on a one‐way ANOVA. Crosslinking density trends of the gelAGE‐based hydrogels compared to the gelGPE‐based hydrogels (right). Data are reported as mean ± SDs, *n* = 3 independent experiments. Statistical significances are determined based on a one‐way ANOVA. c) Quantitative evaluation of the print line resolution of gelAGE‐based hydrogels (w/o PA) and gelGPE‐based hydrogels (stoichiometrically balanced and in thiol deficit), as a function of the LAP concentration. Data are reported as mean ± SDs, *n* = 5 repeated measurements per group (Figure S3, Supporting Information). Statistical significances are determined based on a two‐way ANOVA. a–c) The significance of *p* is indicated by ^*^ = *p* < 0.05, ^**^ = *p* < 0.01, ^***^ = *p* < 0.001, ^****^ = *p* < 0.0001.

In this study, two strategies were investigated to enhance the print line resolution by tailoring the resin precursor formulations with either gelatin‐grafted alkyne moieties or by simply using PA. The latter are non‐reactive species featuring absorbance at the irradiation wavelength thus interfering with the PI and reducing the light scattering and the overcuring.^[^ ^41^, ^42^ ^]^ Food dyes represent ideal PA as they are cost‐effective, readily available, and cytocompatible.^[^ ^43^ ^]^ The UV–vis spectra of two food dyes tartrazine (λ_max_ = 410 nm) and Allura red AC (λ_max_ = 510 nm) were measured (**Figure** **3**a, left). These PA featured high hydrophilicity, cytocompatibility and for the tartrazine synchronized absorption wavelengths with the 405 nm LOF used for the photopatterning experiments.^[^ ^43^, ^44^ ^]^ Allura red AC was selected as a negative control to highlight the importance of the synchronization of the PA absorbance and the irradiance wavelength.^[^ ^45^ ^]^ The two PA were added to the gelAGE‐based hydrogel precursor solutions and their effects on the photocrosslinking efficiency were assessed using photorheology and measuring the hydrogel sol fraction (mass loss at day 1) values. A time sweep analysis (Figure 3a, center) was performed on the control gelAGE (5%)‐based hydrogel precursor solution featuring PEG4SH (10%) as crosslinker, and LAP (0.1%) as PI. A 0.02% of both tartrazine and Allura red AC was added to the control formulation. As expected, the trends of the storage modulus demonstrated a clear influence of both PA, particularly tartrazine, on the photocrosslinking kinetic of the gelAGE‐based hydrogel formulation. Indeed, during the UV–vis (390–500 nm) light exposure of the precursor solutions the storage modulus of the control formulation showed an almost instant (≈1 s) steep increment, whilst the presence of the PA greatly delayed the photocrosslinking reaction with a steep increment of the elastic modulus only ≈5 s after light exposure. Furthermore, the highly efficient absorbance property of the tartrazine‐laden precursor was remarked by a less pronounced increment, after crosslinking, of its storage modulus with invariably smaller values compared to the Allura red AC‐laden hydrogel formulation.

The sol fraction analysis was performed to quantitively address the photocrosslinking efficacy of the three gelAGE‐based hydrogel precursor solutions (Figure 3a, right). The sol fraction is a quantitative and straightforward analysis of the photocrosslinking efficacy and defines the fraction of the non‐crosslinked polymer washed out of the hydrogel network after 24 h.^[^ ^46^ ^]^ The control hydrogel formulation showed a sol fraction (4.3 ± 3.9%) significantly smaller than the gelAGE‐based hydrogel formulation containing the tartrazine (39.0 ± 3.2%) confirming its high absorbance and effect on the hydrogel photocrosslinking efficacy. Furthermore, the lack of absorbance wavelength synchronism between the LOF and the Allura red AC and thus its poor effect on the photocrosslinking reaction was confirmed by its sol fraction value (1.1 ± 5.3%), which was comparable to the control precursor solutions.

The second strategy adopted to absorb the LED light was based on the introduction of a gelatin‐based hydrogel featuring alkyne groups (gelGPE) which was characterized by light absorbance properties at 405 nm thus representing an ideal alternative to PA for OFAP photopatterning (Figure 3b, left). The photorheological analysis demonstrated the effect of the alkyne moieties on the crosslinking, as indicated by the delayed steep increment of the storage modulus of the gelGPE‐based formulations compared to the gelAGE‐based control (Figure S2, Supporting Information). Furthermore, alkyne moieties can undergo a thiol‐yne click chemistry crosslinking reaction in the presence of a thiolated crosslinker, and a PI.

After complete thiol‐yne reaction, each functional group of the gelGPE was combined with two thiol moieties thus establishing the alkynes as difunctional, instead of the monofunctional allyl groups involved in the thiol‐ene. Indeed, the thiol‐yne click reaction enabled to obtain multifunctional and denser polymeric networks.^[^ ^47^ ^]^ The crosslinking density (ρ_x_) of the gelAGE‐ and gelGPE‐based hydrogel networks was estimated using the theory of rubber elasticity,^[^ ^48^ ^]^ assuming the materials are incompressible (Poisson's ratio (ν) = 0.5), since hydrogels are characterized by extremely high‐water content and show large strain under low loading conditions.^[^ ^49^ ^]^

The gelGPE‐based hydrogel precursor solutions were formulated by using a stoichiometric balance of thiol and alkynes moieties (2 thiols alkyne^−1^) and in thiol deficit (1 thiol alkyne^−1^). In both precursor solutions a 5% concentration of gelGPE with either 2.6 or 5.2% of PEG4SH, and 0.1% of LAP was used. The investigation of different concentrations of thiols in the gelGPE‐based hydrogel formulations was designed to better highlight their effect on the OFAP photopatterning. Indeed, the kinetic of the thiol‐yne reaction is rather correlated to the thiol instead of the alkyne concentration since the chain transfer is the rate‐determining step.^[^ ^50^ ^]^ The estimated ρ_x_ value of the gelGPE‐based hydrogel featuring a thiol deficit was 1.03 ± 0.26 × 10^−4^ m. This was, expectedly, four times smaller compared to 4.40 ± 0.82 × 10^−4^ m of the gelGPE‐based hydrogels with stoichiometrically balanced alkynes and thiols. The gelAGE‐based hydrogel showed a crosslinking density of 6.06 ± 0.11 × 10^−4^ m which was significantly higher than both gelGPE‐based hydrogels (Figure 3b, right). This phenomenon confirmed the high absorbance properties of the gelGPE which theoretically should have yielded a denser polymer network than gelAGE, due to the nature of the thiol‐yne crosslinking mechanism.

Further confirmation of this hypothesis was shown by the sol fraction values (Figure 3a, right) of the gelGPE‐based hydrogel stoichiometrically balanced (36.9 ± 2.7%) and in thiol deficit (28.5 ± 9.1%). These values are indeed in line with those of the tartrazine which already showed to have a synchronized absorption spectrum, as the gelGPE, with the LED of the OFAP set‐up. Moreover, ρ_x_ influences other physicochemical properties, besides mechanical stiffness, such as solute diffusivity.^[^ ^51^ ^]^ Indeed, the swelling trend (Figure 3b, center) of the gelGPE‐based hydrogel in thiol deficit which was characterized by a low crosslinking density showed significantly higher swelling (24.1 ± 4.5%), after 24 h, compared to its stoichiometrically balanced analog (16.5 ± 1.5%) and the gelAGE‐based control (10.8 ± 1.0%).

The OFAP photopatterning resolution for the different screened resins was evaluated and trends were reported (Figure 3c). The resolution of VPP processing technologies can be influenced by physical and chemical parameters such as light scattering and radical diffusion, respectively. This latter parameter is strictly correlated with the crosslinking efficacy and density, although one of the challenges of VPP techniques is the precise confinement of the photocrosslinking reaction in predetermined areas of the resin.^[^ ^52^ ^]^ Indeed, another important factor for the OFAP photopatterning process was the introduction of the resin formulations of PA and alkyne moieties (gelGPE) which limited the overgrowth (light scattering) by absorbing part of the light and challenged the PI. The print line resolution assessment of the photopatterned resin containing 0.1% LAP, showed a significantly more resolved print line for the gelAGE‐based hydrogels featuring PA compared to the control (0.323 ± 0.015 mm). Tartrazine which was characterized by a synchronized absorbance spectrum at 405 nm showed print lines with smaller diameters (0.124 ± 0.007 mm) compared to the effect of the Allura red AC on the gelAGE‐based hydrogel resolution (0.222 ± 0.028 mm). Being characterized by the presence of alkynes groups which showed absorbance at 405 nm, the gelGPE‐based hydrogels were expected to yield more resolved print lines compared to the control. Indeed, the stoichiometrically balanced gelGPE‐based hydrogel featured print line diameters in the order of the tartrazine‐based analogs (0.086 ± 0.030 mm). Furthermore, the gelGPE‐based hydrogel crosslinked in thiol deficit showed a larger print line (0.162 ± 0.009 mm) as expected since the kinetic of the thiol‐yne reaction is strongly correlated to the thiol concentration. The resolution trends of the screened photoresins were invariably higher when the LAP concentration was increased to 0.2%. Nevertheless, similarly to the hydrogels featuring 0.1% LAP, these trends showed a better resolution for the resins containing PA and gelGPE compared to the gelAGE‐based hydrogel control. Contrary to the resins featuring 0.1% LAP, a non‐significant improvement of the resolution from the presence of tartrazine compared to the Allura red AC was noticed, similarly for the gelGPE‐based hydrogel in thiol defect. This phenomenon could be explained by the double increment of PI which overcame the absorbance of the PA and of the alkyne moieties of the gelGPE.

### 2.3 OFAP for the Embedded 3D Printing of Self‐Standing Structures

**Figure 4:**
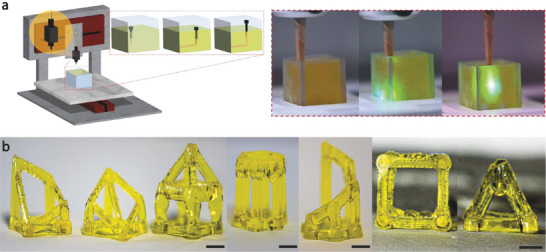
OFAP 3D fabrication of gelAGE‐based constructs. a) Schematic (left) and actual representation (right) of the 3D printing process using the OFAP platform. The LOF is immersed into the photosensitive‐laden vat and its spatiotemporal position is automatically controlled to generate a 3D structure. b) Proof‐of‐concept fabrication of self‐standing 3D structures (e.g., hollow cube and pyramid) based on gelAGE‐hydrogels with tartrazine as a photo absorber. Scale bars: 3 mm.

The first part of this study focused mainly on the understanding of the OFAP setup and a comprehensive investigation of the fabrication parameters, alone and in combination, and their effects on the stability and resolution of the photopatterned structures. Although, the 2D photopatterning approach showed good efficiency in terms of fabrication times, roughly two times faster compared to a DLP printer (Original Prusa SL1S speed), the possible applications remained limited to the XY space relying mainly on the automatized spatiotemporal control of the optical fiber over the surface of a resin‐laden VAT. In this context, the rationale of developing resins based on thiol‐ene click reaction was centered on the fast reaction kinetics and oxygen insensitivity to ensure a sufficient polymerization degree in each point of the resin surface and enough green strength. To unlock the full potential and demonstrate the high flexibility of the OFAP platform, the setup was upscaled from 2D to embedded 3D printing by introducing an LOF, with different physical features as core diameter and NA, in direct contact with gelAGE‐based photosensitive resins. A pivotal aspect of this study was the exploitation of the versatility of the OFAP printing setup toward the 3D freeform fabrication of self‐standing structures. In a proof‐of‐concept approach, the LOF was used to directly print 3D structures within a tartrazine‐laden gelAGE‐based resin bath (**Figure** **4**a; Movie S2, Supporting Information). The OFAP 3D printing process was based on the controlled spatiotemporal position (feed rate = 1 or 2 mm s^−1^) of the tip of the optical fiber in direct contact with the photosensitive resin bath to draw consecutive horizontal, vertical, and diagonal patterns in the 3D space. The gelAGE (5%)‐based precursor formulation used in the 2D photopatterning approach was demonstrated to be a reliable and performing hydrogel suitable for processing with OFAP. Indeed, the physicochemical properties of this precursor formulation showed high crosslinking efficacy and polymerization degree, as indicated by the contained values of sol fraction and swelling. GelAGE was already introduced as resin for stereolithography 3D printing and was characterized by a concentration ≈10 to 20%,^[^ ^53^ ^]^ thus for the OFAP 3D fabrication the gelAGE concentration was increased from 5 to 10%. The gelAGE‐based resin formulation, along with an increment of its concentration, was characterized by a higher LAP content (0.3%). To balance the relatively low 3D printing feed rate, the PI concentration was increased to enhance the radical production per time. Furthermore, the optical fiber used for the 3D printing fabrication featured a different core diameter and numerical aperture (*d* = 0.50 mm, *NA* = 0.63), and was made of polymer instead of quartz. The new material was ideal for the 3D printing approach since the optical fiber is in direct contact with the resin while moving, thus the more brittle and fragile quartz fiber could have been subjected to damage or even breakage. The new polymer optical fiber was characterized by different light intensity outputs spanning, from lowest to highest, from 17 to 130 mW cm^−2^ (well above the maximum intensity obtained with the quartz fiber = 48 mW cm^−2^). The adjustment of the precursor and PI concentration helped to enhance the reactivity of the material during the printing process and the LED irradiance of the new optical fiber was increased to achieve a higher crosslinking rate. The initial focus while developing this new optical fiber‐based 3D printing technique was centered on the fiber tip‐to‐resin interface where a small buildup of cured resin was present. These small parts of cured resin were continuously detached from the optical fiber tip during the movement within the bath, thus yielding print lines featuring slightly bumpy textures. The printing of vertical features was the most straightforward aspect of the 3D fabrication process. Indeed, while driving the optical fiber upward (feed rate = 2 mm s^−1^) within the resin‐laden bath several physical forces were involved particularly at the fiber tip‐to‐resin interface, and helped the continuous removal of cured buildups from the fiber tip. The upward controlled movement of the fiber combined with the buoyancy of the gelAGE‐based resin reservoir facilitated not only the stability of the printed construct but also the detachment of the buildups of crosslinked resin. Moreover, the localized increment of the refractive index in correspondence with the vertical print line acted as an optical constraint, thus favoring the propagation of the patterned front through the resin volume.^[^ ^54^ ^]^ For the fabrication of horizontal print lines, the feed rate was decreased to 1 mm s^−1^ while keeping the LED irradiance constant. Furthermore, the light penetration depth played an important role and required further optimization. This aspect was particularly important when crosslinking horizontal lines, considering the self‐standing nature of the printing constructs. The presence of tartrazine in the precursor solution was ideal to limit the light penetration depth and increase the z resolution of the structures limiting the crosslinking to the desired layer thickness.

Once the optimization of the OFAP 3D printing system was completed on both the vertical and horizontal motions, more complex 3D structures were printed (Figure 4b, right). A cube featuring an 8 mm side with a print line resolution ≈1.7 mm was fabricated. In this structure, the main aspects to optimize were the transition movements from vertical to horizontal paths which resulted in a slightly less resolved edge compared to the main print line. Diagonal paths were introduced in an 8 mm‐side square‐based pyramid which featured print lines with a resolution of ≈1.7 mm and a height ≈8 mm. To demonstrate the flexibility of the structure's design of the OFAP 3D printing platform more complex analogs were fabricated (Figure 4b, left and center). The gelAGE‐based hydrogel precursor formulation was not the only resin tested for the OFAP‐based 3D printing. Indeed, to demonstrate the universality of this platform for the OFAP‐based 3D printing approach, a technical resin based on polyethylene glycol diacrylate (PEGDA) featuring methacrylic moieties was used. The 3D printed constructs, hollow square‐based pyramids, and a hollow cube are shown in Figure S4 (Supporting Information). The structures revealed a print line resolution of ≈1.9 mm. The PEGDA‐based resin precursor formulation included the same concentration of PA and LAP of the gelAGE‐based resin. Nevertheless, a more in‐depth description of the experimental procedure for the PEGDA precursor preparation was reported in Supporting Information.

Taken together, the proof‐of‐concept 3D fabrication based on the OFAP platform showed promising results although highlighting some aspects to further optimize to enhance the quality of the prints.

## 3 Conclusion

This study introduced a novel AM fabrication strategy, OFAP, for the 2D photopatterning of gelatin‐based photosensitive resins using a benchtop printer and a cost‐effective LOF. The developed OFAP setup, integrated with an automated platform and open‐source coding software (Python), allowed for precise control over the spatiotemporal position, irradiance, and exposure time of the LED light source. The holistic investigation of the effects of the OFAP parameters such as gap, vat thickness, and irradiance on the print lines resolution demonstrated a close intercorrelation between these variables. Gap and irradiance were varied to adjust the printing resolution, thus fabricating constructs characterized by progressive elements. The use of tartrazine and Allura red AC as PA in the gelAGE‐based resin precursor proved effective in enhancing the photopatterning resolution. Furthermore, the introduction of the novel gelGPE featuring alkyne moieties exhibited near‐visible UV absorbance properties offering an alternative approach to traditional PA. The versatility of the OFAP platform was demonstrated by its successful transition from photopatterning to freeform 3D printing within a gelAGE‐based resin bath. The ability to directly print self‐standing structures (e.g., hollow cube and squared‐based pyramid) within a low‐viscosity resin bath, to the best of our knowledge, represents the first example of optical fiber‐based embedded 3D printing. The proof‐of‐concept study provided promising results and underscored the flexibility of the OFAP fabrication platform. Nevertheless, to further advance the OFAP 3D platform some improvements can be foreseen as a more engineered optical fiber tip with, for instance, hydrophobic coatings which would prevent the formation of buildups and smoothen the texture of the printed structures. Additionally, an optical fiber featuring a smaller core diameter will enhance the resolution further. The optimization of the coding scripts (Python and G‐code) and the printing parameters (e.g., feed rate) could also lead to the introduction of round features in the printed constructs.

Overall, the OFAP approach offered a facile, reproducible, and versatile solution for photopatterning suggesting the use of OFAP as a straightforward platform to adapt the print line resolution and generate incremental details within structures. Furthermore, OFAP freeform 3D printing of gelatin‐based structures showed potential applications in various fields, including tissue engineering and biomimetic scaffold fabrication.

## 4 Experimental Section

### Synthesis of Functionalized Gelatin with Allyl Moieties (gelAGE)

The gelAGE synthesis was adapted and optimized based on previously published protocols.^[^ ^53^ ^]^ Type A gelatin from porcine skin (Sigma Aldrich) was dissolved in deionized water (10%) for 2 h at 37 °C. Two types of gelAGE were synthesized, G_1MM_ and G_2LH_, with only the latter featuring thermoreversibility. The nomenclature refers to the reaction conditions and the educts molar concentrations as already thoroughly indicated in the previous study.^[^ ^28^ ^]^ Sodium hydroxide (NaOH, 2 m, Sigma Aldrich) in a concentration of 0.4 or 2.0 mmol gram^−1^ of gelatin, and allyl‐glycidyl ether (AGE, Sigma Aldrich) in a concentration of 12 or 24 mmol gram^−1^ of gelatin were added to the gelatin at 65 °C for 1 or 2 h. The deprotonated amino groups on the gelatin backbone were linked to the less substituted carbon of the heterocyclic ring of AGE through nucleophilic substitution. The synthesized gelAGE products were dialyzed (Spectra/Por 6, Fischer Scientific; molecular weight cut‐off = 1 kDa) against deionized water and then lyophilized.

### Synthesis of Functionalized Gelatin with Alkyne Moieties (gelGPE)

The grafting of the alkyne functional groups on the gelatin backbone was inspired by the gelAGE reaction mechanism. Similarly, type A gelatin from porcine skin (Sigma Aldrich) was dissolved in deionized water (10%) for 2 h at 37 °C. NaOH (2 m, Sigma Aldrich) in a concentration 0.4 mmol gram^−1^ of gelatin, and glycidyl propargyl ether (GPE, Sigma Aldrich) in a concentration of 24 mmol gram^−1^ of gelatin were added to the gelatin at 65 °C for 2 h. NaOH induced the alkaline conditions which led to the same nucleophilic substitution that drove the deprotonated amino groups,on the gelatin backbone, to link to the exposed carbon site on the heterocyclic GPE ring. The synthesized gelGPE polymer was dialyzed (Spectra/Por 6, Fischer Scientific; molecular weight cut‐off = 1 kDa) against deionized water and then lyophilized.

### Molecular Characterization of the Modified Gelatin‐Based Biopolymers

Aqueous gel permeation chromatography (GPC) analysis (0.1 m NaNO_3_, 0.02 wt.% NaN_3_) of gelAGE and gelGPE was conducted using a Malvern system (Malvern) (Figure S5, Supporting Information). The apparatus consisted of a Viskotek GPCmax (in‐line degasser, 2 piston‐pump, and autosampler), column oven (35 °C), refractive index (Viskotek), and SEC‐MALS 20 (Viskotek) detector. Column set was 2 × A6000m (length: 300 mm, width: 8 mm, porous polyhydroxy methacrylate polymer, particle size A6000M = 13 µm). The flow rate was 0.7 mL min^−1^. Calibration was performed using polyethylene glycol standards (Malvern). The molecular weight of the PEG standards was comprised between 0.4 to 700 kDa. Samples were allowed to dissolve overnight (3 mg mL^−1^) and filtered through a syringe filter (Thermo Fischer Scientific; 0.45 µm regenerated cellulose) prior to analysis. Proton nuclear magnetic resonance (^1^H‐NMR) spectra of gelAGE and gelGPE were recorded on a Bruker Biospin 400 MHz spectrometer (Bruker) with deuterium hydroxide (D_2_O) as solvent (Figure S6, Supporting Information). As an internal reference, the signal at δ = 4.79 ppm was used. The phenylalanine peaks at δ = 7.45 – 7.25 ppm were used as reference signals for AGE and GPE modifications and calibrated to 5 protons. To determine the degree of modification (DoM) of both functionalized gelatins, the proton integral of the allyl and alkyne moieties was compared to the phenylalanine (Phe). On average, 1 gram of gelatin contains 21 milligrams of phenylalanine (2.1%). The composition of acid‐treated porcine skin gelatin was acquired from the gelatin handbook of the Gelatin Manufacturers Institute of America (GMIA). The DoM was calculated based on Equations ((5), (6), (7)).

(5) $$\begin{array}{ccc} Mw_{\mathrm{Phe},\mathrm{gelatin}} & = & Mw_{\mathrm{Phe}}-Mw_{H_{2}O}=165.19\;g\;\mathrm{mo}l^{-1}-18.01\;g\;\mathrm{mo}l^{-1}\\ & = & 147.18\;g\;\mathrm{mo}l^{-1} \end{array}$$

(6) $$\begin{array}{ccc} n_{\mathrm{Phe},\mathrm{gelatin}} & = & \frac{m_{\mathrm{Phe},\mathrm{gelatin}}}{Mw_{\mathrm{Phe},\mathrm{gelatin}}}=\frac{0.021\;g}{147.18\;g\;\mathrm{mo}l^{-1}}\\ & = & 0.143\;\mathrm{mmol}\;\mathrm{per}\;\mathrm{gram}\;\mathrm{of}\;\mathrm{gelatin} \end{array}$$

(7) $$n_{\mathrm{allyl},\mathrm{gelatin}}=n_{\mathrm{Phe},\mathrm{gelatin}}\times \int \mathrm{allyl}\;\mathrm{or}\;\mathrm{alkyne}\;\mathrm{hydrogen}$$

### Photoresin Precursor Solutions Preparation

The freeze‐dried gelAGE (G_1MM_) and gelGPE together with a commercially available PEG4SH (JenKem Technologies), in a powder state, were dissolved in phosphate buffer saline (PBS, 1‐fold, Sigma Aldrich). Eventually, the photoinitiator LAP (Sigma Aldrich) was diluted into the resin precursor (0.1 or 0.2%) from a freshly prepared stock solution (1%) in PBS. The resin precursor solutions were allowed to thoroughly dissolve at 37 °C for a few minutes before the transfer in a custom‐made vat. Furthermore, the PA tartrazine (Sigma Aldrich) and Allura red AC (Sigma Aldrich) were diluted into the gelAGE‐based precursor (0.02%) from a freshly prepared stock solution (0.5%) in PBS. The gelAGE (G_2LH_) featuring thermoreversibility was used only for the fabrication of the multilayer structure exploiting its physicochemical properties.

### Physicochemical Characterization of gelAGE‐ and gelGPE‐Based Hydrogels

The swelling and mass loss analysis of both gelAGE‐ and gelGPE‐based photoresins was adapted from previous studies.^[^ ^55^, ^56^, ^57^ ^]^ In brief, cylindrical hydrogels (diameter: 6 mm, height: 1 mm) were fabricated. Samples were weighed after the crosslinking to obtain the initial mass (m_initial,t0_) and then lyophilized to get the dry weight (m_dry,t0_). The other samples (*n* = 6) were incubated at 37 °C in PBS for 24 h. Samples were blotted dry and weighed to obtain the swollen mass (m_swollen,t_) and lyophilized to get the dry weight (m_dry,t_).

The swelling ratio (*q*) of the hydrogels was calculated based on Equation (8).

(8) $$q=\frac{m_{\mathrm{swollen},t}}{m_{\mathrm{dry},t}}$$

The percentage of sol fraction which corresponds to the amount of non‐crosslinked polymer that leaches out from the hydrogel network after 24 h, was calculated based on Equation (9).

(9) $$\%\;\mathrm{sol}\;\mathrm{fraction}=\frac{m_{\mathrm{dry},\mathrm{initial}}-m_{\mathrm{dry},t}=24h}{m_{\mathrm{dry},\mathrm{initial}}}\times 100$$

Thereby, *m*_dry,initial_ was calculated using the actual macromer fraction as reported in Equation (10 and 11).

(10) $$\text{actual macromer fraction}=\frac{m_{\mathrm{dry},t0}}{m_{\mathrm{initial},t0}}$$

(11) $$m_{\mathrm{dry},\mathrm{initial}}=m_{\mathrm{initial}}\times \text{actual macromer fraction}$$

### Mechanical Properties and Crosslinking Density Measurements

The mechanical properties of the hydrogels were measured in an unconfined, uniaxial compression test, using a dynamic mechanical analyzer (BOSE, ElectroForce 5500 system). Cylindrical samples after crosslinking (diameter: 5 mm, height: 3.5 mm, *n* = 3) were subjected to a strain ramp until 25% compression of the sample height was reached (0.01 mm s^−1^, preload buffer time 20 s). The Young's modulus (*E*) was calculated from the slope of the stress‐strain curve. *E* was used for the estimation of the sample crosslinking density using the theory of rubber elasticity (Equation 12) assuming that the material was incompressible (ν = ½).^[^ ^50^ ^]^

(12) $$\rho_{x}=2\left(1+v\right)\frac{E}{RT}$$

### UV–vis Spectroscopy and LED Irradiance Measurements

The UV–vis analysis of both functionalized gelatin biomaterials and the photo absorbers was conducted with a spectrometer (Genesys 10S UV–vis, Thermo Scientific). The samples were dissolved in distilled water and loaded on a transparent glass vial. First, a blank was obtained using only distilled water (solvent) and subsequently, each sample was measured within a wavelength spectrum spanning from 200 to 800 nm. A radiometer (Radiometer RMD, Opsytec Dr. Gröbel) was used to measure the irradiance. The optical fiber was placed at 0.15 mm from the measuring sensor and a live monitoring of the light intensity was performed.

### Confocal Microscopy

gelAGE‐based hydrogel precursor solutions were stained with fluorescent (FITC; MW: 500 kDa; Sigma Aldrich and Texas red; MW: 70 kDa; Thermo Fischer) dyes‐labeled high molecular weight dextran. For imaging the scaffolds were placed in a Petri dish with a glass bottom slide (FluoroDish Cell Culture Dish, World Precision Instruments, FD35‐100) containing a few drops of water. The imaging was conducted using a confocal reflection microscope (Leica LSM SP8, Leica Microsystem) with a laser wavelength of 496 and 561 nm, respectively.

### Rheological Measurements

Rheological properties of the different resin formulations were characterized using an Anton Paar MCR 702 rheometer (Anton Paar) with a 25 mm parallel plate geometry at a 500 µm gap. A solvent trap was used to limit the evaporation phenomenon during the experiments. All measurements were conducted by loading the freshly prepared hydrogel precursors on the lower plate at room temperature (21 °C). Photorheological measurements (time sweep) were performed by coupling the rheometer with a UV–vis light source (Dr. Hönle, bluepoint 4) equipped with a flexible light guide. The wavelength spectrum was narrowed using an optical filter between 390 and 500 nm. The glass of the lower enabled the UV–vis light to crosslink the precursors using an exposure time of 10 s at 7.5 cm distance (light probe to the lower plate; light dosage = 254 mJ cm^−2^).

### Vat Fabrication

The vats were designed with Autodesk Fusion 360 software and exported as.stl files. They were further processed into.sl1s files using the Prusa Slice software. Afterward, the vats were printed using the Original Prusa SL1S Speed DLP printer. Post fabrication, the vats were cleaned and post‐cured both for 5 min exposure in a UV chamber CW1S and with 2000 flashes in a light curing unit (Otoflash G171, NK‐Optik). The vat dimensions were tailored depending on the type of fabrication process. The photopatterning experiments were conducted in a vat with a 21.25 mm side, an overall thickness of 5 mm, and an inner thickness of 0.20 mm able to contain 60 µm of photoresin precursor solution. The 3D printing was conducted instead in a larger vat with a side of 18 mm and a thickness of 20 mm enabling the printing of relatively large 3D structures.

### Setup for OFAP Photopatterning and 3D Printing

A benchtop and compact (56 × 106 × 166 mm) continuous and high‐power output LED‐light source (Silver LED, 405 nm, Prizmatix) connected with a quartz‐based optical fiber featuring a 0.25 mm diameter and an NA of 0.66 was used for the photopatterning of click resins. The optical fiber was placed onto a 3D printer (3DDiscovery, RegenHU) printhead. The LOF was controlled via the Prizmatix Multi LED Ctrl software and connected to a computer, as it was the 3D printer which was controlled via the integrated 3D Discovery software. Python was used to connect and synchronize both software. In this way, the whole printing process was programmed and controlled using phyton. To ensure an ideal positioning of the vat containing the resin precursor solution an FDM‐printed holder was mounted on the collector plater of the 3D printer. The feed rate was used at 1 and 2 mm s^−1^. For the OFAP 3D printing a different optical fiber made of polymer and featuring a diameter of 0.50 mm and an NA of 0.63 mm was used in the aforementioned setup, although using a different vat.

### Multilayer Photopatterning

The thermoreversibility properties of the G_2LH_ type of gelAGE were used to fabricate a two‐layer structure. A first layer of G_2LH_ was evenly spread onto the surface of a vat with a 0.50 mm inner thickness, then physically gelled by incubating the vat in a custom‐made cooling chamber for a few minutes. The first layer was then photopatterned and cooled down to gel the unreacted resin again. Subsequently, another layer of G_2LH_ was distributed onto the first material surface and then photopatterned. Eventually, the construct was thoroughly rinsed with distilled water to remove the excess of unreacted resin.

### Fiber Resolution Assessment

Images of the photopatterned structures designed for the print line resolution assessment were taken with a stereomicroscope (Leica DMS1000) using constant lighting conditions and focus. Each image was processed using the open‐source software Fiji. Briefly, the images were cropped and duplicated. A threshold (Red‐Yen) was applied to the original images which was subsequently eroded and dilated. Eventually, the image calculator feature was used to sum the two images and obtain a smooth and clear image to accurately calculate the print line diameter in different spots of the designed photopatterned structure (Figure S3, Supporting Information).

### Statistical Analysis

All data were expressed as means ± standard deviations (SDs) for *n* ≥ 3. The normal distribution of the data was checked with a Shapiro‐Wilk test. GraphPad Prism software (GraphPad software) was used to perform a one‐ or two‐way analysis of variance (ANOVA) with a Dunnett's or Tukey's post hoc multiple comparison test, respectively, to determine statistical significance. Values of *p* < 0.05 were considered statistically significant. ^*^ = statistically significant differences (^*^ = *p* < 0.05, ^**^ = *p* < 0.01, ^***^ = *p* < 0.001, ^****^ = *p* < 0.0001).

## Conflict of Interest

The authors declare no conflict of interest.

## Supporting information

Supporting Information

Supplemental Movie 1

Supplemental Movie 2

## Data Availability

The data that support the findings of this study are available from the corresponding author upon reasonable request.
