# Supplementary material for: Optical Fiber‐Assisted Printing: A Platform Technology for Straightforward Photopolymer Resins Patterning and Freeform 3D Printing
Source: Adv Sci (Weinh). 2024 Jun 21;11(32):2403049. doi: 10.1002/advs.202403049 (PMC11348141; doi:10.1002/advs.202403049)
Supplement: Supplementary file 1 — Supporting Information [file ADVS-11-2403049-s002.docx]

**Supplementary information**

Supplementary Information for

**Optical Fiber-Assisted Printing: A Platform Technology for Straightforward Photoclick Resins Patterning and Freeform 3D Printing**

Alessandro Cianciosi, Maximilian Pfeiffle, Philipp Wohlfahrt, Severin Nürnberger and Tomasz Jungst*

*Corresponding author email: tomasz.juengst@uni-wuerzburg.de

**This pdf file includes:**

Supplementary Methods

Supplementary Figures 1 to 6

**Preparation of the PEGDA-based resin precursor formulation for OFAP-based 3D printing**:

The technical resin precursor formulation was based on commercially available PEGDA (700 Mn, Sigma-Aldrich). The PEGDA was firstly scaled directly in a specific container for a planetary vacuum mixer (THINKY Mixer, THINKY, USA). Subsequently, stock solutions of LAP (1%) and tartrazine (0.5%) were dissolved in distilled water and added to the PEGDA to obtain their final concentrations of 0.3% and 0.02%, respectively. The final PEGDA concentration of 50% was adjusted by adding distilled water. The precursor solution was mixed for 10 min at 1000 rpm in the planetary vacuum mixer. During preparation and until the printing process, the PEGDA-based resin was protected from light with aluminum foil.


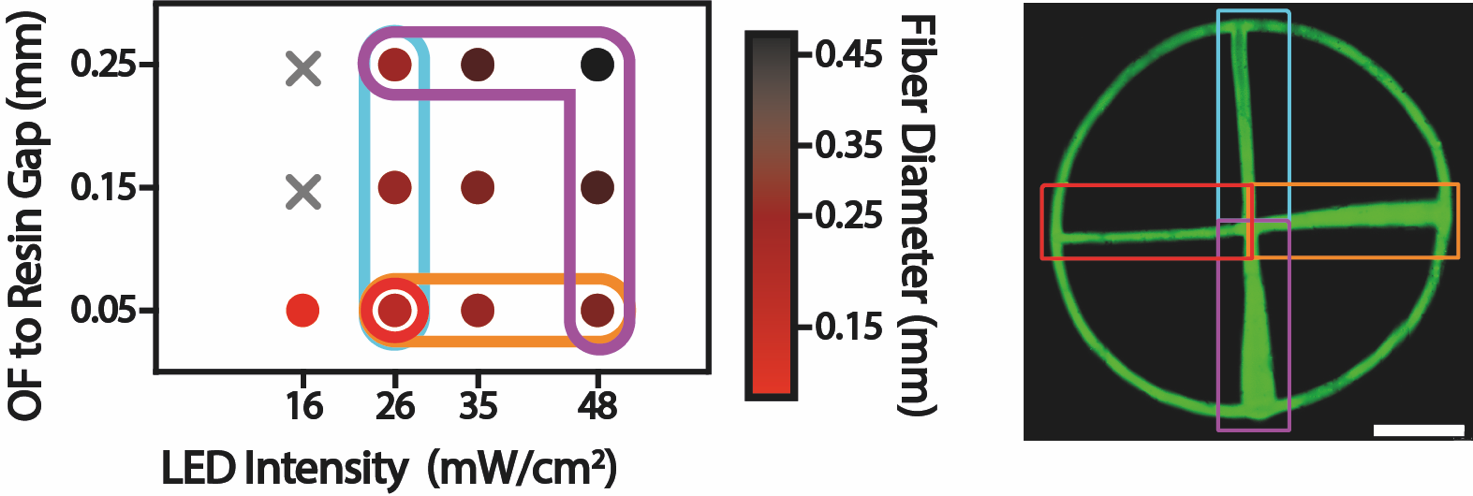


**Supplementary Figure 1.** Photopatterned structure characterized by 4 different print lines fabricated by real-time adjustment of the OFAP parameters, as irradiance and gap. Different colors highlighted the different real-time OFAP parameter adjustments with the relative print line resolution, and their effect on the patterned structure with progressive features. Scale bar = 3 mm.


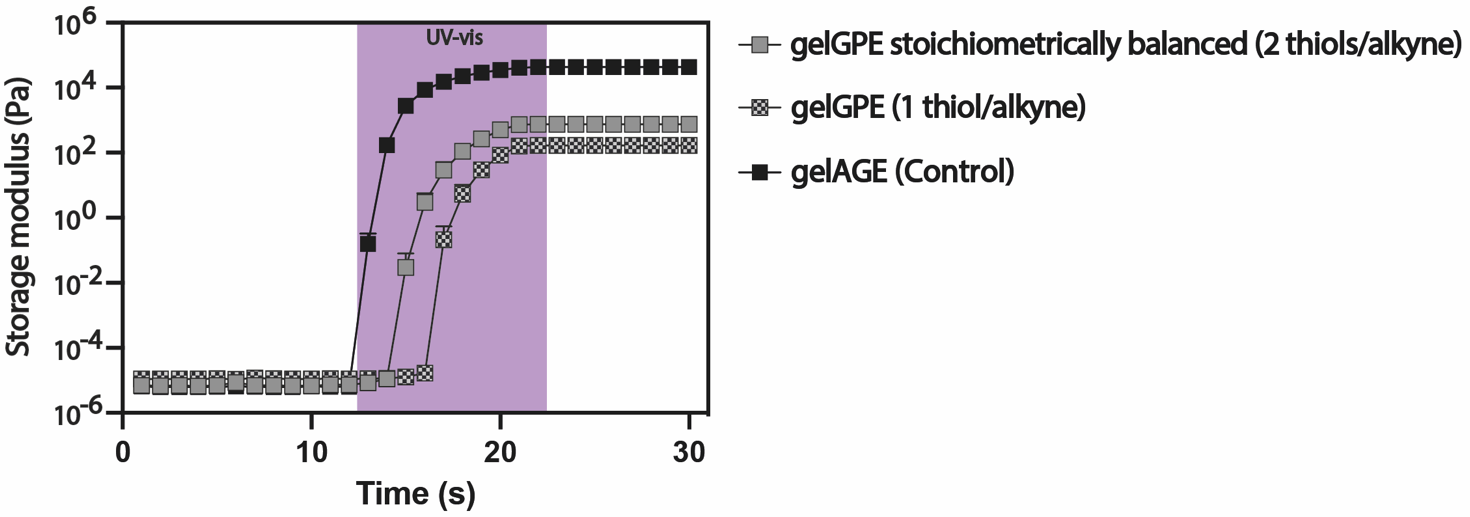


**Supplementary Figure 2.** Photorheological analysis of gelGPE-based precursor solutions. Photo-rheological oscillatory analysis (n = 3, 10 rad s^-1^ oscillation frequency, 10% shear strain): Time sweeps trends of the gelAGE- (control) and gelGPE-based precursor with different stoichiometric balance (1 and 2 thiols/alkyne).


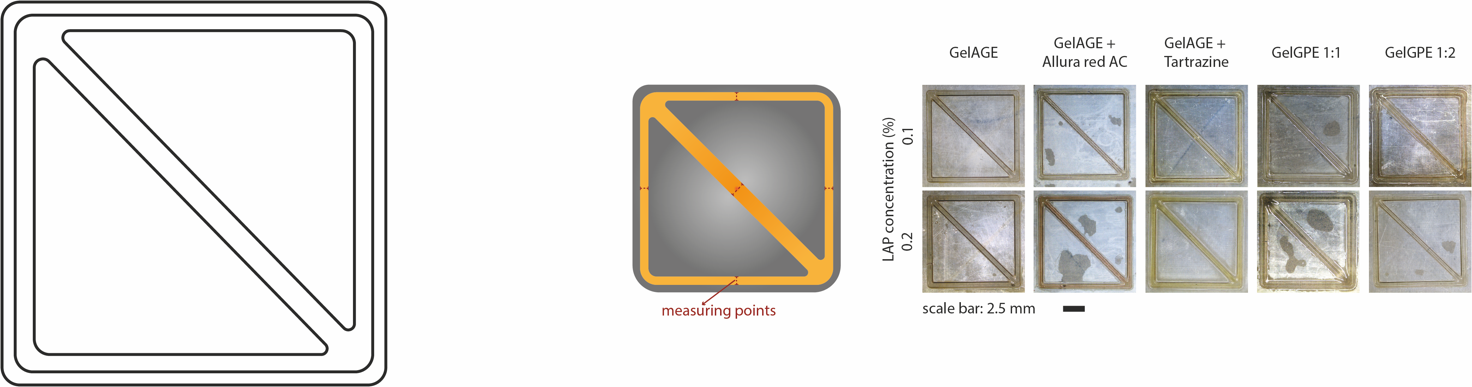


**Supplementary Figure 3.** Fiber resolution assessment of different photopatterned resins. Schematic of the designed pattern for the determination of the fiber diameter (left). Schematic of the selected measuring areas (n = 5) within the photopatterned structures along with the original stereomicroscope images of different photosensitive resins (e.g., gelAGE w/o PA and gelGPE with different molar ratios).

**
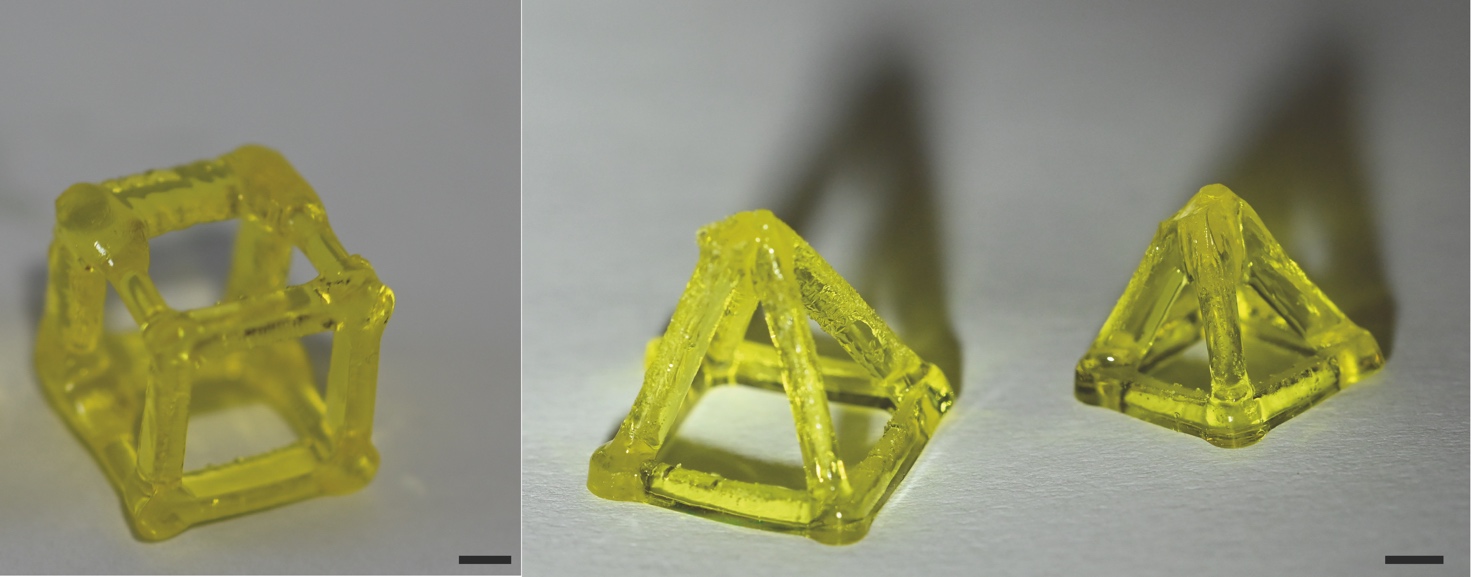
**

**Supplementary Figure 4.** OFAP 3D fabrication of PEGDA-based constructs. Proof-of-concept fabrication of self-standing 3D structures, hollow cube on the left side, and square-based pyramids with two different sizes. Scale bars: 3 mm.


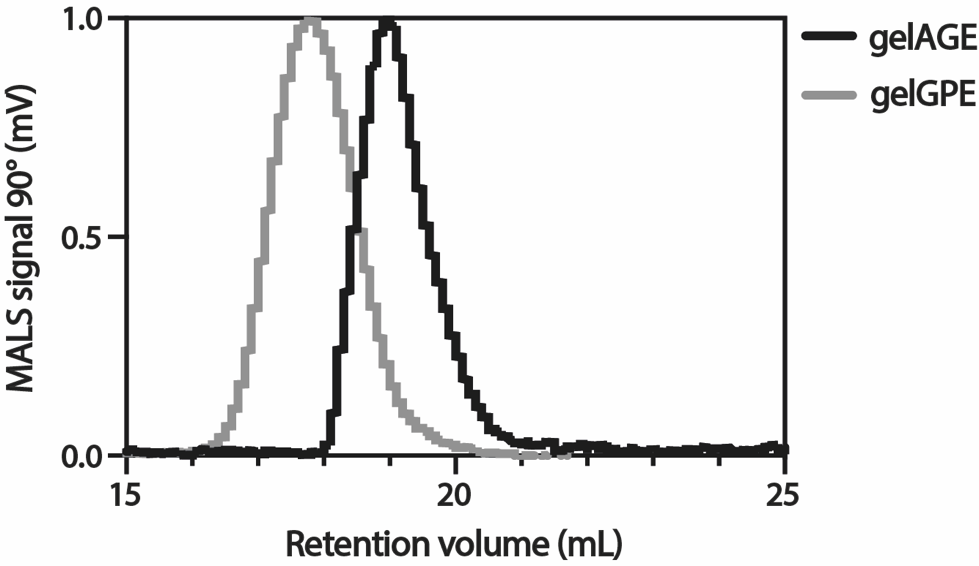


**Supplementary Figure 5.** Aqueous-GPC spectra comparison of gelAGE and gelGPE. Multi angle light scattering (90°-MALS) chromatogram of gelAGE (G_1MM_) in comparison to gelGPE.


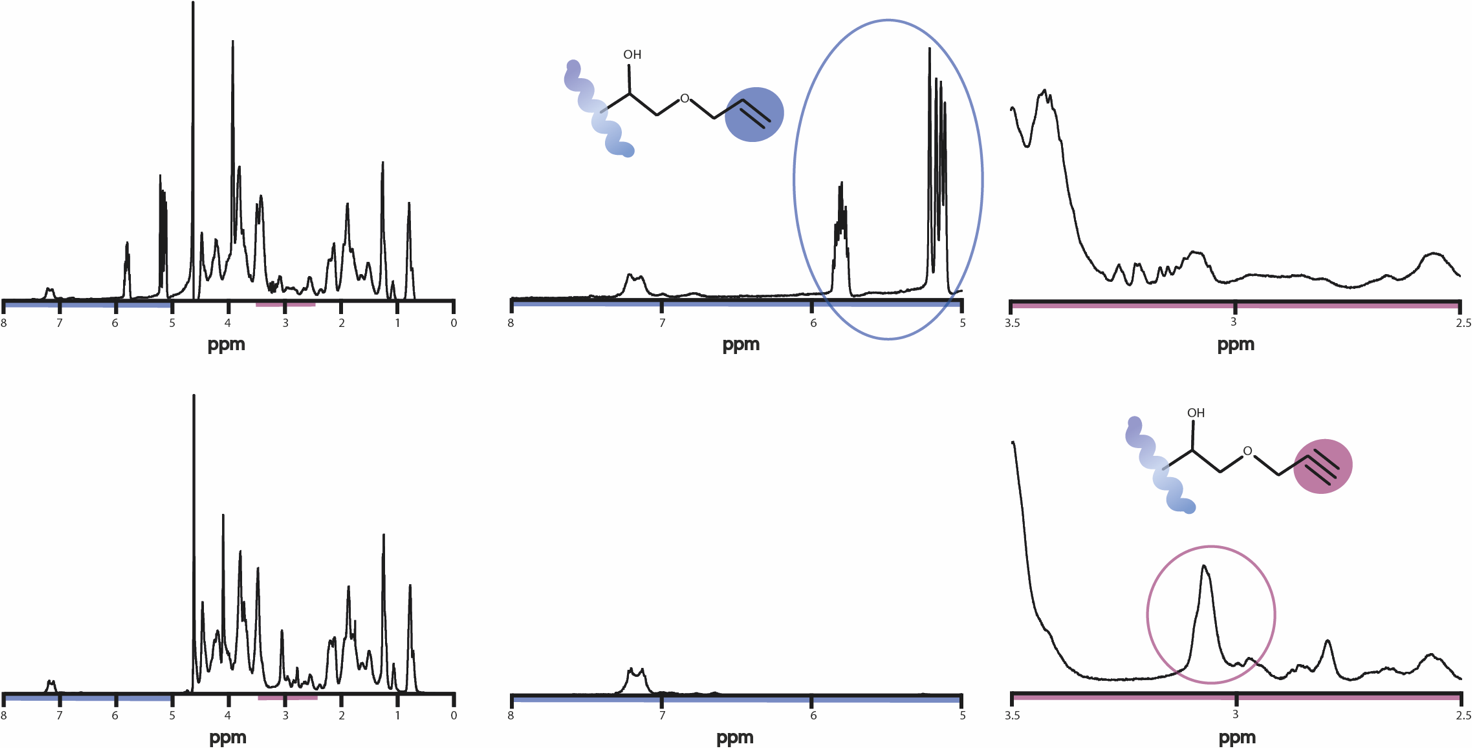


**Supplementary Figure 6.** ^1^H-NMR spectra comparison of gelAGE and gelGPE. ^1^H-NMR spectrum of a gelAGE (top) and gelGPE sample (bottom). The blue highlighted area of the spectra (δ = 7.8 – 5.0 ppm) contains the phenylalanine peak (δ = 7.45—7.25 ppm) and its integral is normalized for its 5 protons. Two other peaks (δ = 6.0—5.0 ppm) are representing the allyl protons of the gelAGE. The integral value of the single proton (δ = 6.02 – 5.88 ppm) is used to determine the DoM of the gelAGE. The peak centered around δ = 3.15—3.05 ppm refers to the carbon-carbon triple bond grafted on the gelGPE, and its integral is used to determine the DoM of the gelGPE.
